# Supplementary material for: Identifying surface reaction intermediates with photoemission tomography
Source: Nat Commun. 2019 Jul 18;10:3189. doi: 10.1038/s41467-019-11133-9 (PMC6639300; doi:10.1038/s41467-019-11133-9)
Supplement: Supplementary file 1 — Supplementary Information [file 41467_2019_11133_MOESM1_ESM.pdf]

Supplementary Information - Identifying surface reaction intermediates with photoemission tomography

Xiaosheng Yang,[1,2,3] Larissa Egger,[4] Philipp Hurdax,[4] Hendrik Kaser,[5] Daniel Lüftner,[4] François C. Bocquet,[1,2] Georg Koller,[4] Alexander Gottwald,[5] Petra Tegeder,[6] Mathias Richter,[5] Michael G. Ramsey,[4] Peter Puschnig,[4] Serguei Soubatch,[1,2] and F. Stefan Tautz[1,2,3]

[1] *Peter Grünberg Institut (PGI-3), Forschungszentrum Jülich, 52425 Jülich, Germany*
[2] *Jülich Aachen Research Alliance (JARA), Fundamentals of Future Information Technology, 52425 Jülich, Germany*
[3] *Experimental Physics IV A, RWTH Aachen University, 52074 Aachen, Germany*
[4] *Institute of Physics, University of Graz, NAWI Graz, 8010 Graz, Austria*
[5] *Physikalisch-Technische Bundesanstalt (PTB), 10587 Berlin, Germany*
[6] *Physikalisch-Chemisches Institut, Ruprecht-Karls-Universität Heidelberg, 69120 Heidelberg, Germany*

Supplementary Methods: Geometry of photoemission measurements

Photoemission experiments were conducted with the toroidal analyser [1]. The sample is located in vertical orientation on the symmetry axis of the analyser (Supplementary Figure 1). We use p-polarized ultraviolet light, with photon energies from 20 to 45 eV, passing through the entrance slit of the analyser. The k-maps shown in the paper were measured with the photon energy of 35 eV, corresponding to the strongest molecular emission in this experiment. Only photoelectrons emitted in the incidence plane indicated grey in Supplementary Figure 1 pass through the entrance slit of the analyser to enter the energy dispersive elements (toroids) [1]. With the angle of incidence of 40° and at the photon energy of 35 eV, the major emissions from the frontier molecular states are found in the emission angle range of 40° to 50° (θ angle and k-vector in the figure) and thus close to the light polarization vector **A**. In this geometry the plane wave final state approximation is mathematically equivalent to the independent atomic centers approximation (IAC) [2].

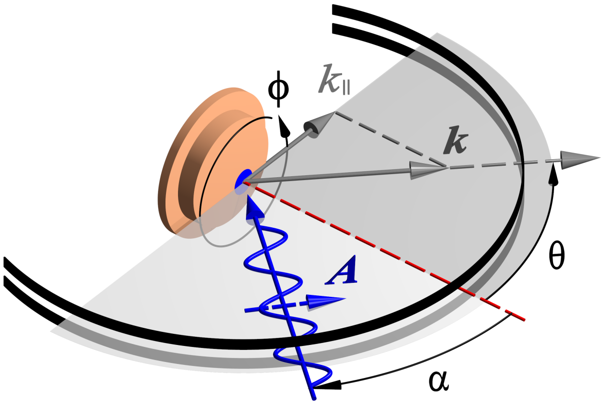

Supplementary Figure 1: Schematic geometry of the photoemission experiment. p-polarized light, with the polarization vector **A**, enters through the aperture slit and is incident on the sample at an angle α. All electrons in the half circle above the sample (take-off angle $\theta = -85°$ to $85°$) containing the plane of incidence are collected simultaneously. The crystal azimuth (ϕ) is rotated in steps of 1°. Note that only the forward emission direction ($\theta > 0$, dark grey area) is used for the construction of the momentum maps, where $k_{\|} = \sqrt{2mE_{\mathrm{kin}}/\hbar^2}\,\sin\theta$.

Supplementary Note 1: As-deposited DBBA on Cu(110)

The electronic structure calculations for DBBA monolayers adsorbed on Cu(110) are performed in the repeated slab approach, using the epitaxial matrix $\begin{pmatrix} 4 & 0 \\ 1 & 6 \end{pmatrix}$ [3]. The metallic substrate is modeled by five Cu layers with lattice parameter $a_{\mathrm{Cu}} = 3.61$ Å and a vacuum layer of at least 15 Å. All structures discussed below have been locally relaxed, but the atomic positions in the three Cu-layers at the bottom of the slab have been frozen.

For the as-deposited DBBA/Cu(110), we have considered three possible adsorption geometries. Structure #1 depicted in Supplementary Figure 2 is identical to the adsorption geometry suggested by Simonov et al. [3] for which we find an adsorption energy of −3.27 eV. Azimuthally rotating the molecules by 90° such that the zig-zag edges of the molecule are aligned perpendicular to the Cu-rows results in structure #2 which is shown in Supplementary Figure 3. It is energetically more favourable and exhibits an adsorption energy of −3.60 eV. Finally, an adsorption geometry with intact DBBA (Br atoms attached to the molecule) denoted as structure #3 is displayed in Supplementary Figure 4. It turns out to be the least favourable structure with an adsorption energy of −2.27 eV. Supplementary Table 1 summarizes the total energies and adsorption energies of the three structures introduced above. Adsorption energies have been calculated as $E_{\mathrm{ad}} = E_{\mathrm{tot}} - (E_{\mathrm{tot,\ DBBA}} + E_{\mathrm{tot,\ Cu(110)\text{-}slab}})$.

In addition to images of the adsorption geometries, Supplementary Figures 2–4 also show the densities of states and simulated momentum maps for structures #1, #2 and #3, respectively. Note that none of these three structures can explain the experimentally observed momentum maps in Fig. 3a of the main paper. Photoemission tomography for such three-dimensional species is extremely sensitive to the exact molecular geometry and would require the development of a global search algorithm to identify the as-deposited species. This is beyond the scope of this work.

Supplementary Table 1: Total energies E_{tot} and adsorption energies E_{ad} of as-deposited DBBA on the Cu(110) surface.

Structure	Composition	E_{tot} (eV)	E_{ad} (eV)
DBBA (gas phase)	$C_{28}H_{16}Br_2$	−315.01658	
Cu(110)-slab	Cu_{120}	−478.44739	
DBBA/Cu(110) structure #1	$Cu_{120}C_{28}H_{16}Br_2$	−796.73309	−3.269
DBBA/Cu(110) structure #2	$Cu_{120}C_{28}H_{16}Br_2$	−797.05866	−3.595
DBBA/Cu(110) structure #3	$Cu_{120}C_{28}H_{16}Br_2$	−795.73354	−2.270

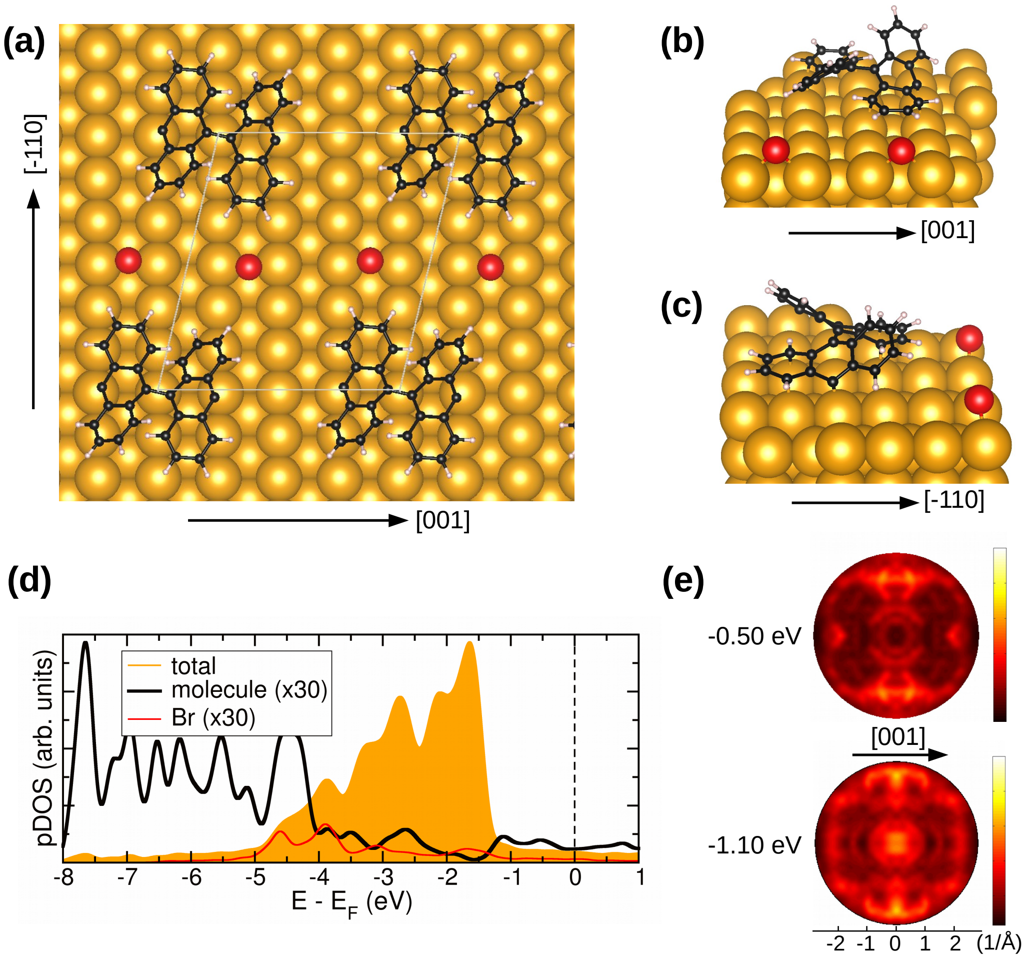

Supplementary Figure 2: DBBA adsorption structure #1 (proposed by Simonov et al. [3]) Panels (a), (b), and (c) show top and side views, respectively, of the relaxed adsorption structure. Panel (d) displays the total density of states (DOS) and the projected DOS (pDOS) for the molecule (black line) and the Br atoms (red line). Panel (e) shows simulated ARUPS momentum maps at binding energies of −0.50 and −1.10 eV, respectively.

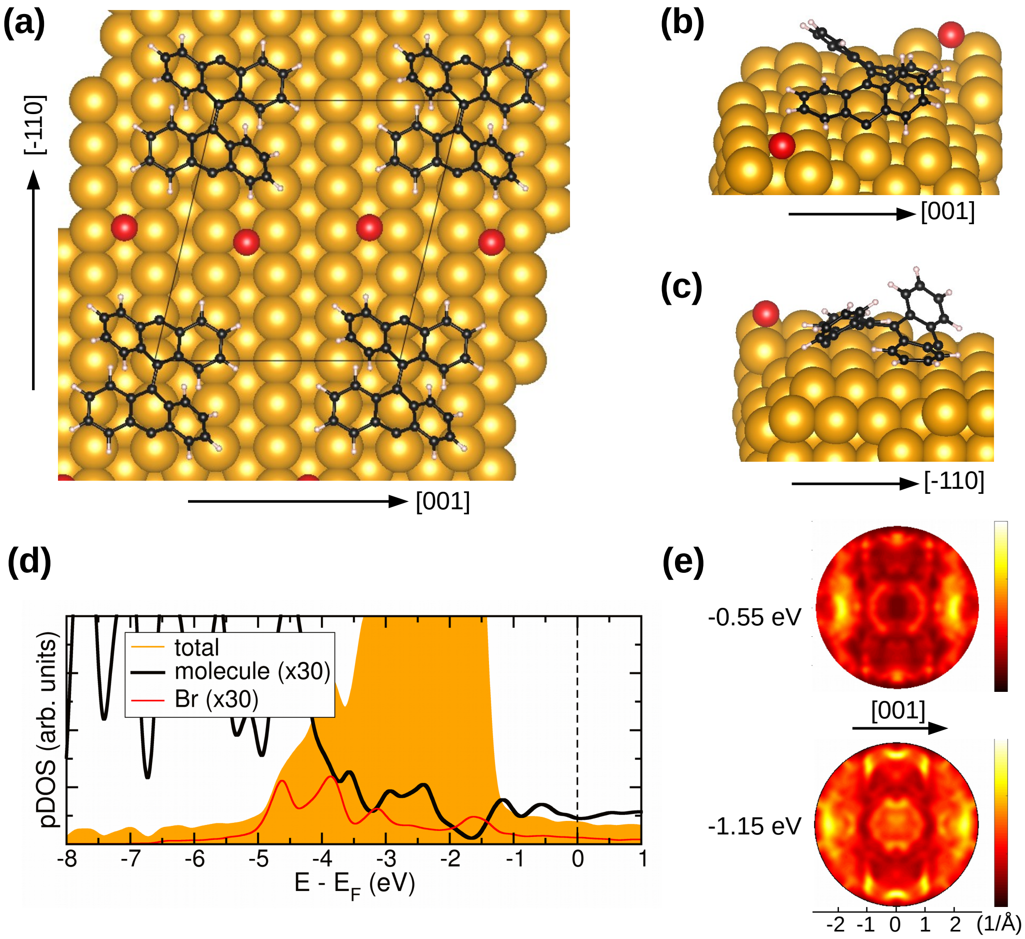

Supplementary Figure 3: DBBA adsorption structure #2. Panels (a), (b), and (c) show top and side views, respectively, of the relaxed adsorption structure. Panel (d) displays the total density of states (DOS) and the projected DOS (pDOS) for the molecule (black line) and the Br atoms (red line). Panel (e) shows simulated ARUPS momentum maps at binding energies of −0.55 and −1.10 eV, respectively.

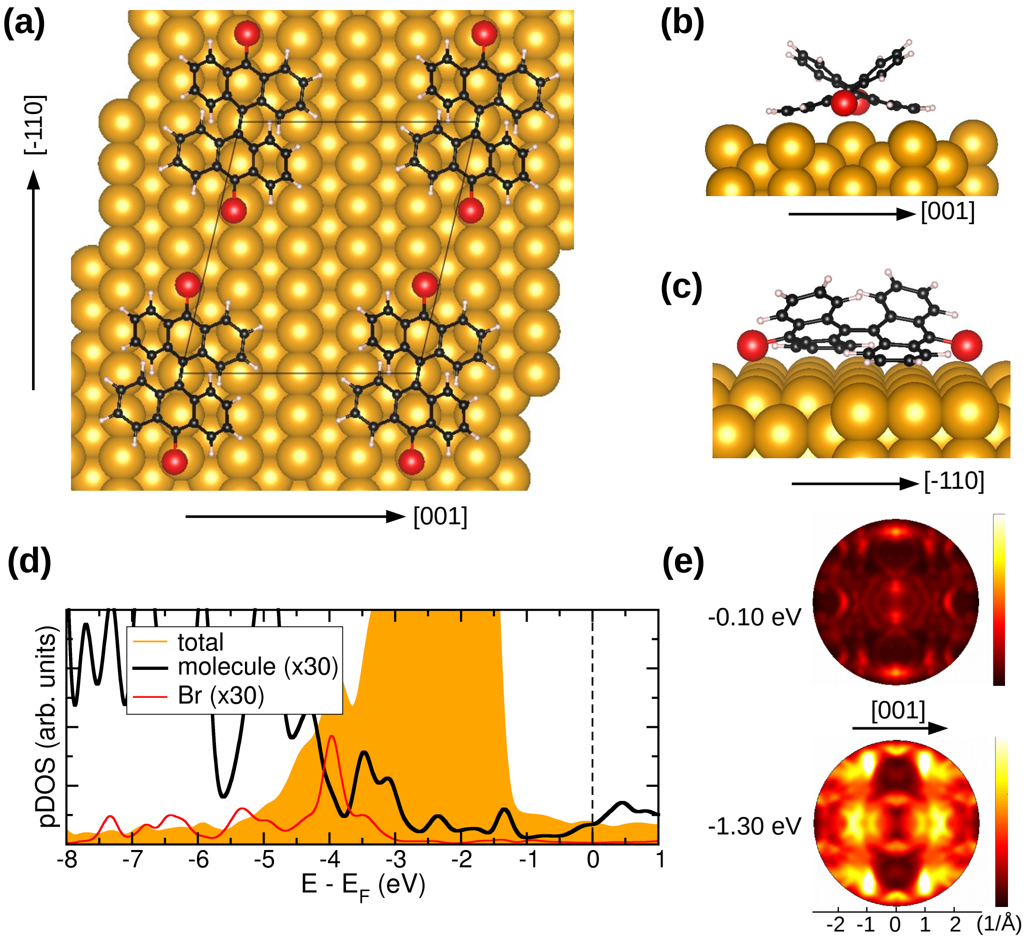

Supplementary Figure 4: DBBA adsorption structure #3. Panels (a), (b), and (c) show top and side views, respectively, of the relaxed adsorption structure. Panel (d) displays the total density of states (DOS) and the projected DOS (pDOS) for the molecule (black line) and the Br atoms (red line). Panel (e) shows simulated ARUPS momentum maps at binding energies of -0.10 and $-1.30\,\mathrm{eV}$, respectively.

Supplementary Note 2: Reaction intermediate on Cu(110)

The electronic structure calculations for reaction intermediates on Cu(110) are performed in the repeated slab approach, now using the epitaxial matrix $\begin{pmatrix} 4 & 0 \\ 2 & 5 \end{pmatrix}$. The metallic substrate is modeled by five Cu layers with lattice parameter $a_{\mathrm{Cu}} = 3.61\,\text{Å}$ and a vacuum layer of at least 15 Å. All structures discussed below have been locally relaxed, but the atomic positions in the three Cu-layers at the bottom of the slab have been frozen.

Supplementary Table 2 summarizes the total energies and adsorption energies of all candidates for reaction intermediates investigated in this work. Adsorption energies have been calculated as $E_{\mathrm{ad}} = E_{\mathrm{tot}} - (E_{\mathrm{tot,\ gas\ phase\ molecule}} + E_{\mathrm{tot,\ Cu(110)\text{-}slab\ +\ 2Br}})$. In particular, we have considered the following three molecular species: (i) the fully hydrogenated bisanthene ($C_{28}H_{14}$), (ii) the partially zig-zag-edge-dehydrogenated $C_{28}H_{12}$ and (iii) the fully zig-zag-edge-dehydrogenated $C_{28}H_8$. For each molecular species, four high-symmetry adsorption sites have been considered, namely the hollow (H), the long bridge (LB), the short bridge (SB) and the top (T) sites. Bisanthene favours the short bridge, yielding an adsorption energy of −5.26 eV, while $C_{28}H_{12}$ and $C_{28}H_8$ both prefer the top site with adsorption energies of −8.01 eV and −12.72 eV, respectively. Note that the latter value agrees with the adsorption energy given by Simonov et al. for $C_{28}H_8$ [3]. Importantly, however, as outlined in the paper, the chemical reaction energies which also take into account the energy necessary to detach hydrogen atoms from DBBA clearly show that bisanthene is the most likely reaction intermediate.

Supplementary Figures 5–7 depict the most favourable adsorption geometries, the densities of states and simulated momentum maps for bisanthene ($C_{28}H_{14}$), $C_{28}H_{12}$ and $C_{28}H_8$, respectively.

Supplementary Table 2: Total energies E_{tot} and adsorption energies E_{ad} of reaction intermediates on the Cu(110) surface at hollow (H), long bridge (LB), short bridge (SB) and top (T) sites.

Structure	Composition	Site	E_{tot} (eV)	E_{ad} (eV)
Cu(110)-slab	Cu_{100}		−398.32704	
Cu(110)-slab + 2Br	$Cu_{100}Br_2$		−405.31483	
bisanthene (gas phase)	$C_{28}H_{14}$		−305.62265	
bisanthene/Cu(110) + 2Br	$Cu_{100}Br_2C_{28}H_{14}$	H	−714.48437	−3.547
bisanthene/Cu(110) + 2Br	$Cu_{100}Br_2C_{28}H_{14}$	LB	−713.83354	−2.896
bisanthene/Cu(110) + 2Br	$Cu_{100}Br_2C_{28}H_{14}$	SB	−716.19721	−5.260
bisanthene/Cu(110) + 2Br	$Cu_{100}Br_2C_{28}H_{14}$	T	−714.97503	−4.038
$C_{28}H_{12}$ (gas phase)	$C_{28}H_{12}$		−292.65724	
$C_{28}H_{12}$/Cu(110) + 2Br	$Cu_{100}Br_2C_{28}H_{12}$	H	−702.88498	−4.913
$C_{28}H_{12}$/Cu(110) + 2Br	$Cu_{100}Br_2C_{28}H_{12}$	LB	−702.56425	−4.592
$C_{28}H_{12}$/Cu(110) + 2Br	$Cu_{100}Br_2C_{28}H_{12}$	SB	−705.49124	−7.519
$C_{28}H_{12}$/Cu(110) + 2Br	$Cu_{100}Br_2C_{28}H_{12}$	T	−705.98567	−8.014
$C_{28}H_8$ (gas phase)	$C_{28}H_8$		−266.79873	
$C_{28}H_8$/Cu(110) + 2Br	$Cu_{100}Br_2C_{28}H_8$	H	−681.81636	−9.703
$C_{28}H_8$/Cu(110) + 2Br	$Cu_{100}Br_2C_{28}H_8$	LB	−683.06676	−10.953
$C_{28}H_8$/Cu(110) + 2Br	$Cu_{100}Br_2C_{28}H_8$	SB	−684.63227	−12.519
$C_{28}H_8$/Cu(110) + 2Br	$Cu_{100}Br_2C_{28}H_8$	T	−684.83779	−12.724

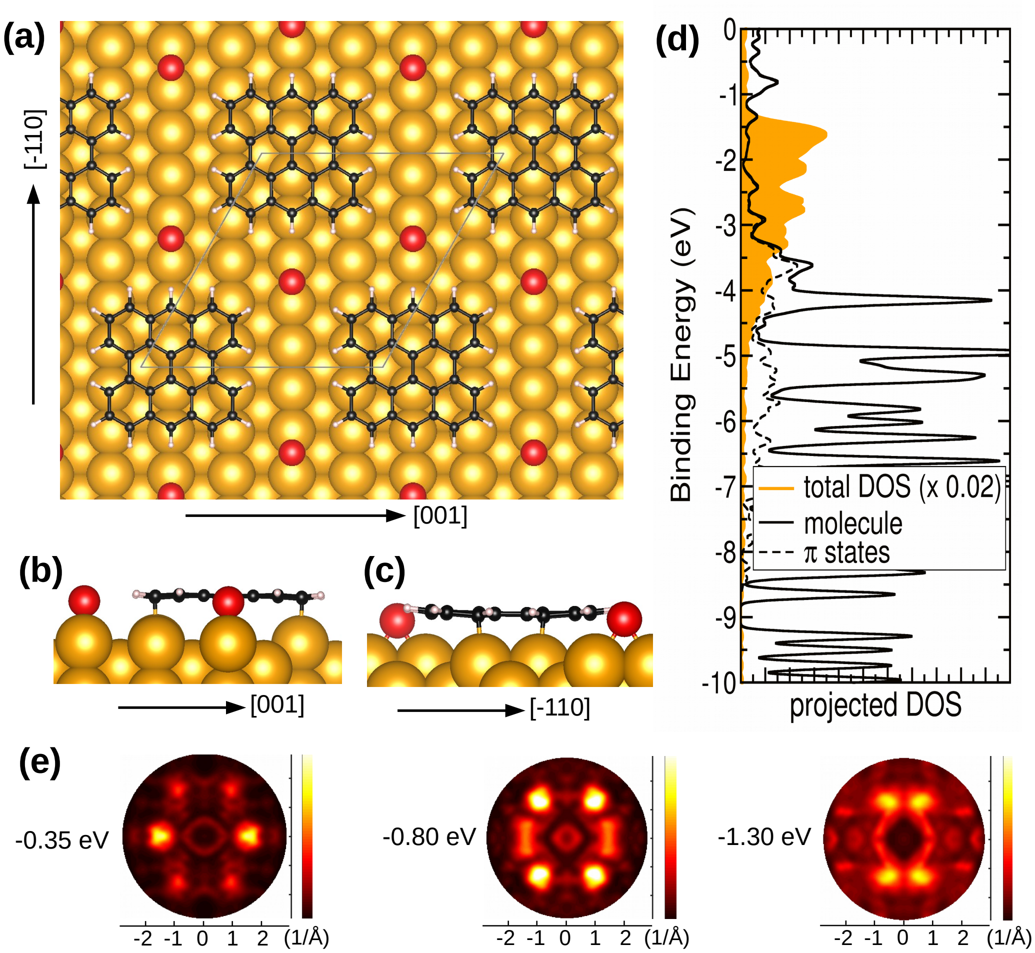

Supplementary Figure 5: Energetically most favourable adsorption structure of bisanthene ($C_{28}H_{14}$)/Cu(110) + 2Br. Panels (a), (b), and (c) show top and side views, respectively, of the relaxed adsorption structure. Panel (d) displays the total DOS (orange) and the projected DOS (pDOS) for the molecule (black line) and molecular π-states (black, dashed line). Panel (e) shows simulated ARUPS momentum maps at binding energies of -0.35 eV, -0.80 eV and -1.30 eV, respectively.

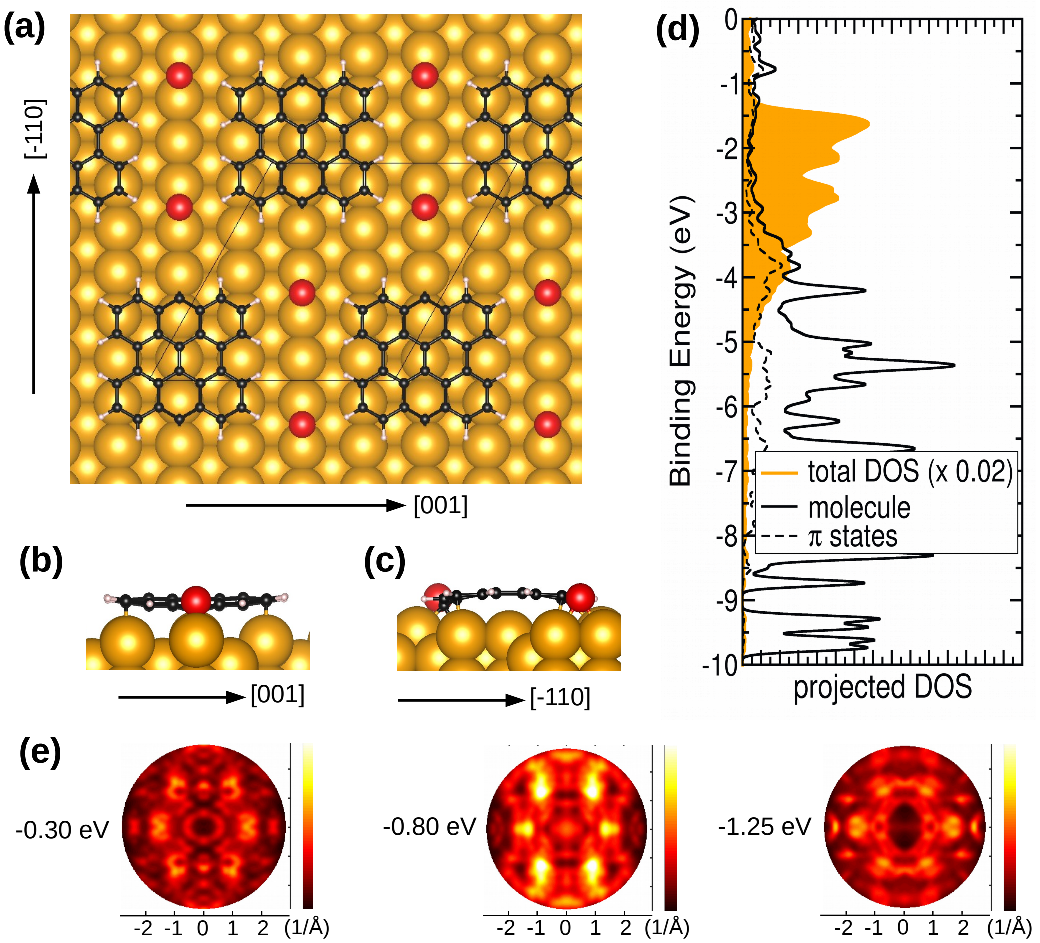

Supplementary Figure 6: Energetically most favourable adsorption structure of $C_{28}H_{12}$/Cu(110) + 2Br. Panels (a), (b), and (c) show top and side views, respectively, of the relaxed adsorption structure. Panel (d) displays the total DOS (orange) and the projected DOS (pDOS) for the molecule (black line) and molecular π-states (black, dashed line). Panel (e) shows simulated ARUPS momentum maps at binding energies of $-0.30\,$eV, $-0.80\,$eV and $-1.25\,$eV, respectively.

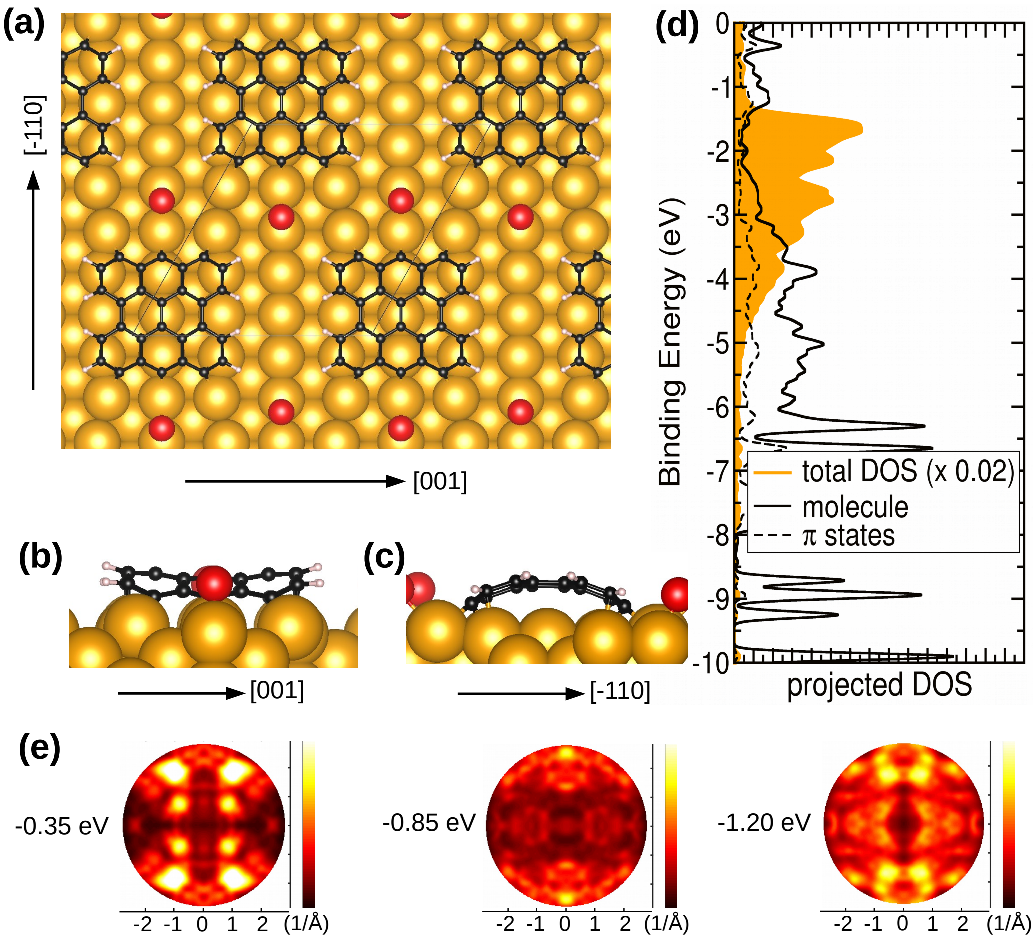

Supplementary Figure 7: Energetically most favourable adsorption structure of $C_{28}H_8$/Cu(110) + 2Br. Panels (a), (b), and (c) show top and side views, respectively, of the relaxed adsorption structure. Panel (d) displays the total DOS (orange) and the projected DOS (pDOS) for the molecule (black line) and molecular π-states (black, dashed line). Panel (e) shows simulated ARUPS momentum maps at binding energies of -0.35 eV, -0.85 eV and -1.20 eV, respectively.

Supplementary Note 3: Simulation of scanning tunneling microscopy contrast

Simulations of scanning tunneling microscopy (STM) contrast were carried out in the framework of the Tersoff-Hamann approximation [4] using the results of DFT calculations as described in Supplementary Note 2 for the Br + $C_{28}H_{14}$/Cu(110), Br + $C_{28}H_{12}$/Cu(110) and Br + $C_{28}H_8$/Cu(110) interfaces. Simulated constant-current mode images are shown in Supplementary Figure 8. Note that Supplementary Figure 8d (left panel, −0.35 V), corresponding to the case of $C_{28}H_{14}$/Cu(110), resembles the STM data of Simonov et al. best (cf. Figure 7a in Ref. [3]).

It is noteworthy that at the bias of −0.1 V (cf. caption of Figure 7a in Ref. [3]), Simonov et al. observe an STM contrast that resembles the shape of the lowest unoccupied molecular orbital (LUMO) of $C_{28}H_{14}$ and based on their DFT results assign the corresponding electronic level to the third state below the Fermi level (HOMO-2) of $C_{28}H_8$ on Cu(110). This assignment implies two additional occupied levels (HOMO and HOMO-1) to be located in a narrow binding energy interval of 0.1 eV. This is ruled out by our photoemission tomography results showing that the former LUMO of $C_{28}H_{14}$ is the closest occupied molecular state to the Fermi level.

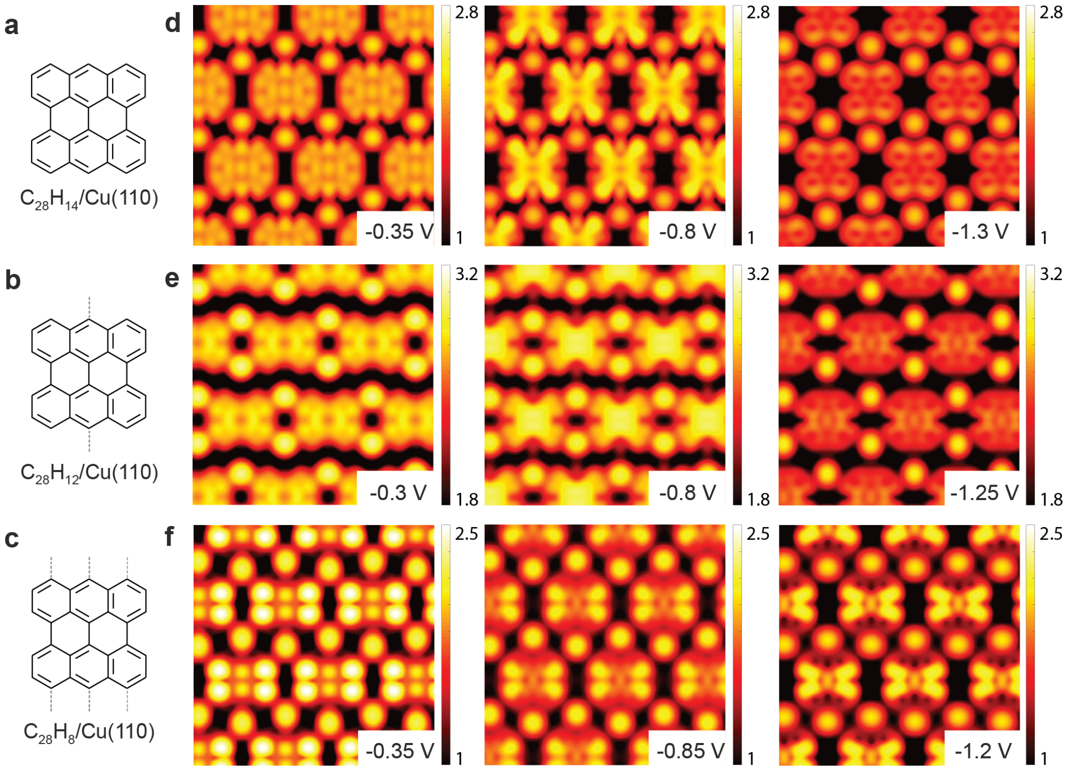

Supplementary Figure 8: Simulated constant-current STM images (a-c) Chemical structure of intermediates and (d-f) simulated constant-current STM images for (d) Br + $C_{28}H_{14}$/Cu(110), (e) Br + $C_{28}H_{12}$/Cu(110) and (f) Br + $C_{28}H_8$/Cu(110) interfaces. Bias voltages are shown in the inset. The colour-code reflects the height of the tip (in Angstrom) above the surface.

Supplementary Note 4: X-ray photoelectron spectroscopy of the reaction intermediate

Supplementary Figure 9 shows the C 1s soft XPS spectrum measured after annealing of DBBA on Cu(110) at 250 °C and fitted with two- and three-component models. The areas under the component lines correspond to the stoichiometry of $C_{28}H_{14}$ for the two-component fitting model (Supplementary Figure 9a) and $C_{28}H_8$ for the three-component fitting model (Supplementary Figure 9b). The relative binding energies of the components of the two- and three-component models are fixed following the models of Simonov et al. [3] for graphene nanoribbons on Cu(111) (cf. Figure 6c in Ref. [3]) and nanographene on Cu(110) (cf. Figure 6b in Ref. [3]), respectively. We note that there is no apparent difference in the fitting quality between the two models, which reveals the restricted capability of XPS to resolve differences in the exact chemical state of the reaction intermediate in question.

The measurements were carried out at the I09 beamline of Diamond Light Source (United Kingdom) using 500 eV photons and a Scienta EW4000 electron analyser. We thank Tien-Lin Lee, Pardeep Kumar Thakur, Anja Haags, You-Ron Lin and Nafiseh Samiseresht for experimental support during this experiment. We thank Diamond Light Source for access to beamline I09.

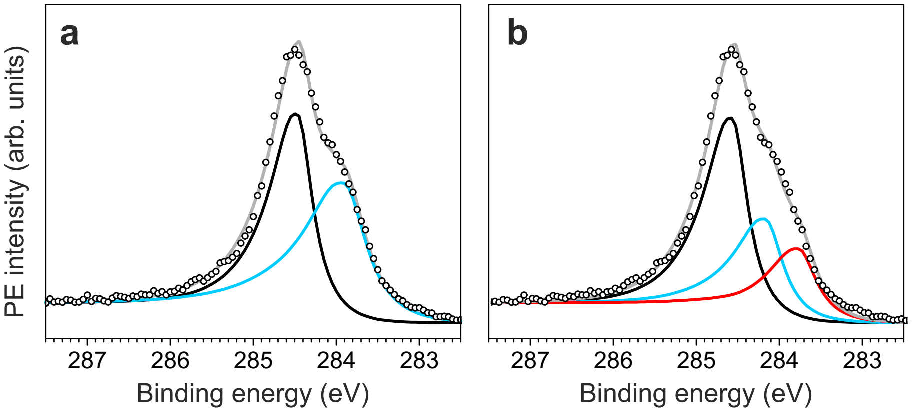

Supplementary Figure 9: XPS of reaction intermediate and fitting models Fitting of the XPS spectrum measured at 500 eV photon energy using (a) two- and (b) three-component models according to Simonov et al. [3]. White dots represent experimental data (identical in both panels). Black, cyan and red curves correspond to, respectively, C1, C2 and C4 components following Ref. [3], i.e., carbon atoms with three neighbouring carbons (C1), hydrogenated carbon atoms (C2) and carbon atoms bonded to copper substrate (C4). Grey curves are the fitting envelop.

Supplementary References

[1] Broekman, L. et al. First results from a second generation toroidal electron spectrometer. *J. Electron Spectrosc. Relat. Phenom.* **144–147**, 1001–1004 (2005).
[2] Puschnig, P. et al. Reconstruction of molecular orbital densities from photoemission data. *Science* **326**, 702–706 (2009).
[3] Simonov, K. A. et al. From graphene nanoribbons on Cu(111) to nanographene on Cu(110): Critical role of substrate structure in the bottom-up fabrication strategy. *ACS Nano* **9**, 8997–9011 (2015).
[4] Tersoff, J. & Hamann, D. R. Theory and application for the scanning tunneling microscope. *Phys. Rev. Lett.* **50**, 1998–2001 (1983).
